# Nonoperative manipulative reduction with Chinese herbs for the treatment of a displaced olecranon fracture: A case report

**DOI:** 10.1097/MD.0000000000010818

**Published:** 2018-05-25

**Authors:** Yu-Pei Chen, Yi-Shan Lan, Wen-Long Hu, Yu-Chiang Hung

**Affiliations:** a Department of Chinese Medicine, Tainan Hospital, Ministry of Health and Welfare; b School of Medicine; c School of Law, National Cheng Kung University, Tainan; d Department of Chinese Medicine, Kaohsiung Chang Gung Memorial Hospital and School of Traditional Chinese Medicine, Chang Gung University College of Medicine; e Kaohsiung Medical University College of Medicine; f Fooyin University College of Nursing; g School of Chinese Medicine for Post Baccalaureate, I-Shou University, Kaohsiung, Taiwan.

**Keywords:** Chinese medicine, displaced olecranon fracture, herbs, manipulative reduction

## Abstract

**Rationale:**

Displaced olecranon fracture is a common injury following a fall or direct trauma to the elbow. There have been no reports of patients with a displaced olecranon fracture who have only received nonoperative manipulative reduction with Chinese herbs.

**Patient concerns:**

The patient was a 64-year-old woman with a complex elbow injury that occurred in a traffic accident. The patient complained of severe, painful limitation of motion on straightening or bending.

**Diagnoses:**

The patient was diagnosed with a displaced fracture of the left olecranon (type IIA olecranon fracture according to the Mayo classification system).

**Interventions:**

The patient underwent nonoperative manipulation with Chinese herbs.

**Outcomes:**

The fracture was successfully reduced. After 3 to 4 months of follow-up, severe pain and disability in the elbow were improved following reduction of the left olecranon fracture in which there was no longer a displacement.

**Lessons:**

Nonoperative manipulative reduction performed by a well-trained physician with Chinese herbs may be a treatment option for displaced olecranon fractures.

## 1 Introduction

The olecranon of the elbow is a trochoid joint articulated with the ulna and humerus bones. Olecranon fractures are common injuries, comprising around 10% of upper extremity fractures in adults.^[1,2]^ Simple, displaced Mayo type II olecranon fractures account for 73.5% to 85% of all olecranon fractures.^[1,2]^ Open reduction with internal fixation has long been accepted as the optimal treatment for displaced olecranon fractures.^[1,2]^

To date, there have been no reports of displaced olecranon fractures treated exclusively with Chinese medicine in the English literature. Here, we report a case of left olecranon fracture with displacement treated with traditional Chinese medicine (TCM).

## 2 Case report

A 64-year-old woman with a history of hypertension and arrhythmia presented to the emergency room with severe pain and immobility in her left elbow, which resulted from a previous traffic accident. The patient complained of severe, painful limitation of motion on straightening or bending of the elbows, and her left hand was heavily bruised and swollen. Physical examination using palpation revealed burning and local tenderness. X-ray revealed a displaced fracture of the left olecranon with soft tissue swelling. The fracture was defined as a type IIA olecranon fracture according to the Mayo classification system.^[1,2]^ The orthopedic specialist suggested surgical open reduction with internal fixation, but the patient hesitated under the consideration of increased surgical risk due to her history of hypertension and arrhythmia. Therefore, the orthopedic surgeon fixed her left elbow with protective clothing only. Later that day, she visited our outpatient clinic to seek help from TCM.

In the first visit, we used the TCM methods for manipulative reduction of the fracture after physical examination. The physician held the patient's left palm in one hand and held the left elbow of the patient in the other hand. Next, the doctor pushed the proximal end of the patient's elbow with his finger to move closer to the distal end, and at the same time, straightened the patient's elbow slowly. Finally, the patient slowly buckled the elbow to 60°. It was then braced securely. We also asked the patient to avoid flexion and extension activities of the elbow and to fix the elbow with protective clothing for about 1 month, until her fracture had healed. That duration depended on X-ray interpretation to ensure complete union of the fracture. The protective clothing, similar to a triangular scarf, was for fixation and immobilization of the displaced olecranon fracture. The patient removed the protective clothing after about 1 month and then started rehabilitation. At the same time, we prescribed Chinese herbs, namely Jenq Guu Tzyy Jin Dan^[3,4]^ 3 g, Shen Tong Zhu Yu Tang^[5]^ 6 g, Corydalis Rhizoma^[6,7]^ 1 g, Ramulus Mori^[8,9]^ 1 g, *Commiphora myrrha*^[10]^ 1 g, and *Boswellia carteri*^[10,11]^ 1 g total daily dose divided by 3. The patient returned to monthly follow-up visits. We used the same prescription over the 3 to 4 months duration of the treatment. During the course of the treatment, we assessed the patient's condition with X-ray, the Mayo elbow performance score (MEPS),^[12,13]^ and the disabilities of the arm, shoulder, and hand (DASH) score (http://www.orthopaedicscore.com/scorepages/disabilities_of_arm_shoulder_hand_score_dash.html).^[14,15]^ After 3 to 4 months of treatment, pain and disability related to the fracture were improved following healing of the left olecranon fracture in which there was no longer a displacement. No complications resulting from the fracture were observed during the follow-up period.

**Figure 1:**
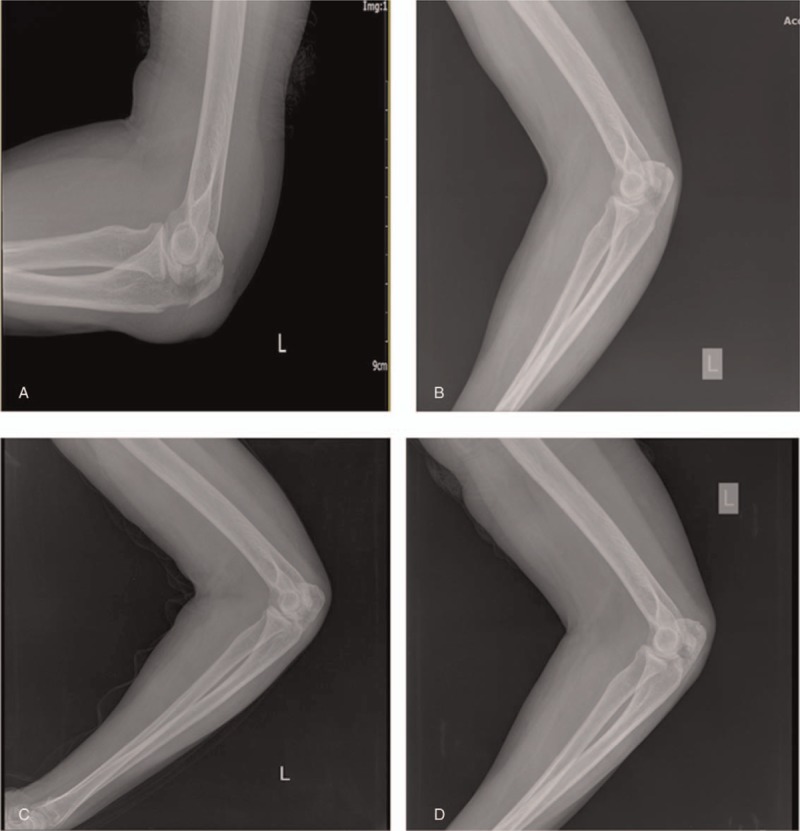
X-ray images showing a displaced fracture of the left olecranon with soft tissue swelling at the initial visit on December 11, 2016 (A) and at the follow-up visits after initiation of treatment on January 5, 2017 (B), February 2, 2017 (C), and March 16, 2017 (D).

**Figure 2:**
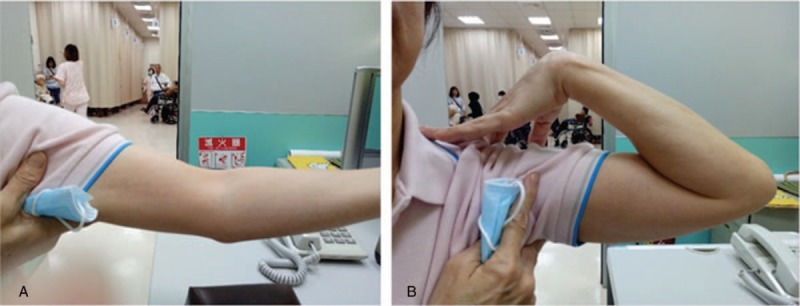
Elbow range of motion at 15° (A) and 140 ° (B) after 4 months of medication on April 4, 2017.

X-ray images taken during the follow-up visits showed that the displaced fracture of the left olecranon with soft tissue swelling improved after manual reduction and medication with Chinese herbs (Fig. 1). The elbow's range of motion ranged from 15° to 140 ° without pain (Fig. 2) and improved from less than 50 ° s (MEPS motion score of 5) to more than 100 ° (MEPS motion score of 20). According to the MEPS score, pain intensity improved from 0 (severe) to 45 (none) points. Stability improved from gross instability (0 points) to stable (10 points). Before treatment, the patient was not able to perform any task that involved the function of the elbow (0 points); after 3 to 4 months of medication, she was able to comb her hair, feed herself, perform hygiene tasks, dress a shirt, and put on shoes (25 points). Overall, the MEPS score improved significantly from 5 to 100 points (χ^2^ = 123.92, *P* < .001) (Table 1). Disability improved significantly from inability to no difficulty in performing some activities after treatment (χ^2^ = 66.86, *P* < .001). The severity of symptoms also improved in the last week of follow-up (χ^2^ = 14.11, *P* < .01) (Table 2).

**Table 1:**
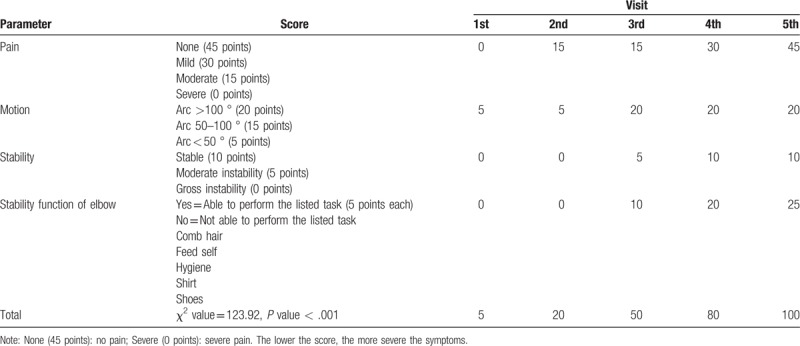
Mayo Elbow Performance Score (MEPS) at different visits.

**Table 2:**
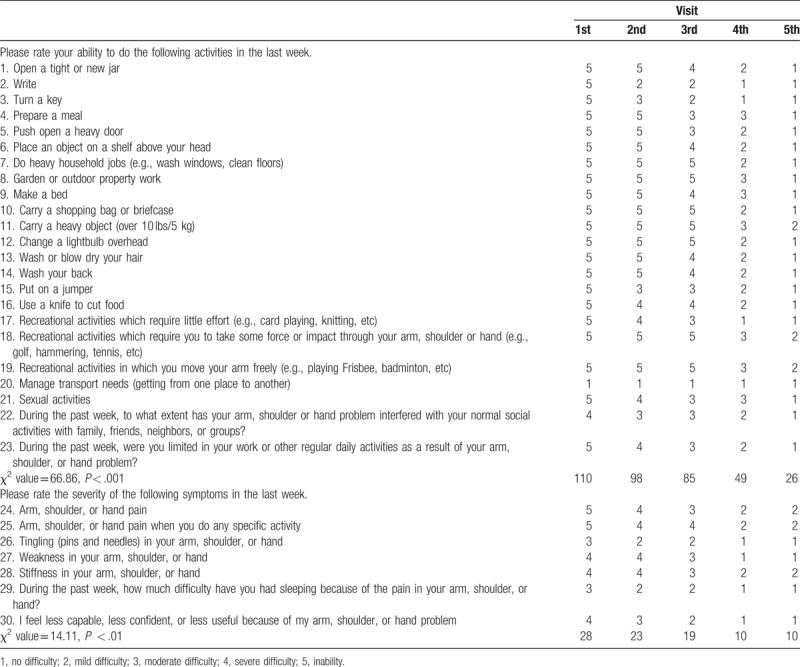
Disabilities of the Arm, Shoulder, and Hand (DASH) score at different visits.

The reporting of this case was approved by the Institutional Review Board of Chang Gung Medical Foundation (IRB permit no. 201800095B0). The report was written after obtaining informed consent from the patient.

## 3 Discussion

Here, we report the case of patient with a displaced Mayo type II olecranon fracture without comminution. The fracture was successfully reduced within 3 to 4 months with TCM, without resorting to surgery. With surgery, the average time of healing of distal humerus olecranon fractures is 3.5 months.^[16]^ If the fracture is not indicated for surgery, patients are at a very high risk of delayed union or nonunion fracture, and elbow function may be severely affected.^[17]^

Elbow function is more likely to be affected after olecranon fracture. Elbow instability and fracture morphology may be prognostic factors for elbow function and arthrosis recovery.^[18]^ The more complex the fracture pattern, the more difficult the elbow mobility. Our patient refused orthopedic surgery. Nonoperative management may be another treatment option for displaced olecranon fractures.^[17,19]^ TCM is the most common alternative medicine used in Taiwan and China. This patient received TCM manual reduction of the fracture with Jenq Guu Tzyy Jin Dan prescription.

The aims of olecranon fracture treatment are to achieve stable bone formation; to attain concentric, stable reduction of the elbow; and to permit early motion. After 3 to 4 months of treatment, the patient exhibited stable bone formation, good reduction of the elbow fracture, and early motion following healing, without displacement. Jenq Guu Tzyy Jin Dan and Shen Tong Zhu Yu Tang complex improves blood circulation, resolves ecchymosis, relieves pain, and reduces local swelling in patients with fractures.^[3–5]^ Corydalis Rhizoma reduces mild to moderate pain and exerts anti-inflammatory effects by decreasing the expression of tumor necrosis factor (TNF)-α, caspase 6, interleukin (IL)-6, and IL-1β.^[6,7]^ Ramulus Mori possesses anti-inflammatory and analgesic activities to suppress IL-6 production by blocking the leukotriene B4 receptor 2-dependent NADPH oxidase 1 reactive oxygen species cascade and inhibit nitric oxide (NO) production by diminishing the expression of inducible nitric oxide synthase (iNOS).^[8,9]^*Commiphora myrrha* and *Boswellia carteri* reduce pain in musculoskeletal disorders and inhibit formalin-induced paw edema, thus revealing its anti-inflammatory and analgesic properties.^[10,11]^ In the case reported herein, treatment with Chinese herbs probably contributed to improve the displaced olecranon fracture.

In addition to medication with Chinese herbs, TCM orthopedic manipulative practice also contributed to displaced olecranon fracture reduction and improved elbow function. Our patient accepted manipulative reduction by a professional TCM orthopedic doctor and the correct position of the elbow was confirmed radiologically before fixation. However, it should be noted that there are some dangerous advanced fractures or displacement with injudicious use of manipulative reduction and tui-na practices.^[20]^ Manipulative reduction and fixed practices must be performed by a well-trained physician.

There are no selection criteria for fracture patients to receive TCM treatment, as all fracture cases are compatible with TCM treatment. Postoperative patients can also be treated with Chinese medicine, which can accelerate fracture healing. In conclusion, our experience showed that TCM medication with manipulative reduction practice and a Chinese herbal compound can improve healing of a displaced olecranon fracture and promote the recovery of elbow function and stability. To our knowledge, this is the first report of a case of a displaced olecranon fracture that was successfully treated with TCM. A randomized, double-blinded controlled trial is needed to evaluate the effectiveness of TCM practices and medication for displaced olecranon fractures in the future.

## Author contributions

YP Chen and YS Lan were responsible for the design and supervision of the study. YP Chen and YC Hung drafted the manuscript. WL Hu and YC Hung participated in the revision of the manuscript and coordination of the study. All authors read and approved the final manuscript.

**Conceptualization:** Yu-Pei Chen, Yi-Shan Lan.

**Data curation:** Yu-Pei Chen, Yi-Shan Lan.

**Investigation:** Yu-Pei Chen.

**Resources:** Yu-Pei Chen.

**Validation:** Yu-Pei Chen, Wen-Long Hu, Yu-Chiang Hung.

**Writing – original draft:** Yu-Pei Chen, Yi-Shan Lan, Yu-Chiang Hung.

**Supervision:** Wen-Long Hu, Yu-Chiang Hung.

**Visualization:** Wen-Long Hu.

**Writing – review & editing:** Wen-Long Hu, Yu-Chiang Hung.

**Project administration:** Yu-Chiang Hung.
